# Effects of *in vitro* azithromycin treatment on bronchial epithelial antiviral immunity in asthma phenotypes

**DOI:** 10.3389/falgy.2025.1605109

**Published:** 2025-06-17

**Authors:** Muzhda Ghanizada, Sofia Malm Tillgren, Louis Praeger-Jahnsen, Nihaya Mahmoud Said, Sisse Ditlev, Helle Frost Andreassen, Nanna Dyhre-Petersen, Samuel Cerps, Asger Sverrild, Celeste Porsbjerg, Lena Uller, Therese Lapperre, Mandy Menzel

**Affiliations:** ^1^ Respiratory Research Unit, Department of Respiratory and Infectious Diseases, Bispebjerg Hospital, Copenhagen, Denmark; ^2^ Unit of Respiratory Immunopharmacology, Department of Experimental Medical Science, Lund University, Lund, Sweden; ^3^ Copenhagen Centre for Translational Research, Copenhagen University Hospital Bispebjerg and Frederiksberg, Copenhagen, Denmark; ^4^ Department of Respiratory Medicine, Antwerp University Hospital, Antwerp, Belgium; ^5^ Laboratory of Experimental Medicine and Paediatrics, University of Antwerp, Wilrijk, Belgium

**Keywords:** asthma exacerbation, antiviral immunity, atopy, rhinovirus, bronchial epithelial cell, azithromycin (AZM)

## Abstract

**Background:**

Azithromycin (AZM) effectively reduces asthma exacerbations and enhances bronchial epithelial cell (BEC) antiviral immunity *in vitro*. However, its clinical impact on different asthma phenotypes is not fully elucidated and differences in treatment response to AZM may be attributable to differences in immune activation to rhinovirus (RV) infection in different inflammatory asthma phenotypes.

**Objectives:**

To explore bronchial epithelial antiviral properties in response to *in vitro* AZM treatment in eosinophilic and non-eosinophilic as well as atopic and non-atopic asthma phenotypes, and to investigate the effects of AZM on the release of RV-induced alarmins and pro-inflammatory cytokines in these asthma phenotypes.

**Methods:**

In this cross-sectional study, we have collected BECs from patients with moderate-to-severe asthma (*n* = 20). The cells were pre-treated with or without 10 µM AZM 24 h before infection with 0.05 MOI RV. Release of IFN-β, IFN-λ, alarmins and pro-inflammatory cytokines were measured 48 h after infection by Mesoscale Discovery (S-plex and U-plex) and then compared across asthma phenotypes, based on blood eosinophils and atopy status.

**Results:**

AZM significantly enhanced IFN-β and IFN-λ protein release in response to RV infection both in eosinophilic and in non-eosinophilic asthma as well as in non-atopic asthma. A less pronounced IFN-β and IFN-λ protein release was also observed in the atopic group. AZM's interferon-inducing effect was, however, largely similar regardless of blood eosinophil count and atopy status. Additionally, AZM prompted the release of TSLP and IL-6 in the non-eosinophilic group only.

**Conclusions:**

Our data suggest that *in vitro*, AZM works primarily by improving bronchial epithelial antiviral resistance by increasing interferons independent of eosinophilia and atopy status, highlighting the broad applicability of AZM in modulating antiviral immunity in asthma as well as the need for identifying predictors of AZM response beyond inflammatory phenotypes.

## Background

Acute asthma exacerbations (AE) are associated with high morbidity and increased healthcare utilization (1). Despite optimal asthma treatment, approximately 5%–10% of patients experience frequent exacerbations (2). Exacerbation is often caused by viral respiratory infection. Moreover, airway inflammation and allergic responses to typical allergens, such as pollen or house dust mites, can further aggravate virus-induced asthma symptoms in atopic individuals (3–6).

Most asthma exacerbations are triggered by rhinovirus (RV) infections (7), and bronchial epithelial cells (BECs) play a crucial role in viral infections by serving both as a protective barrier against invading viruses and orchestrators of the inflammatory response to infection (8). However, the antiviral response of the airway epithelium in asthma may be dysregulated by producing insufficient and/or delayed antiviral interferons such as IFN-β and IFN-λ in response to viral stimuli (9–13). We have previously shown that bronchial epithelial cells from patients with eosinophilic or atopic asthma exhibit an impaired induction of antiviral interferons with increasing disease severity (12). In addition to a dysregulated antiviral immune response, BECs from patients with asthma exhibit an over-production of inflammatory cytokines, promoting eosinophilic inflammation in both atopic and non-atopic asthma (14, 15). Furthermore, the inflammatory response to viruses varies based on the inflammatory phenotype, disease severity, and atopy status (12).

Exacerbation-sparing effects in asthma have been reported for several macrolide antibiotics, such as telithromycin (16), clarithromycin (17) and azithromycin (18, 19), with effects being most well described for azithromycin (AZM). Guidelines have therefore positioned AZM as an add-on treatment option in GINA step 5, as an alternative for biologics or long-acting muscarinic antagonists. However, response to AZM is variable amongst patients and good predictors to guide clinicians are currently lacking. While the benefit of AZM was observed in both eosinophilic and non-eosinophilic phenotypes in one study (18), the effect was only significant in non-eosinophilic patients in another (19). Moreover, whereas most patients included in previous studies were atopic, the effect of atopy status on the response to AZM treatment has not been investigated so far. Thus, investigating how AZM affects T2 (atopy, eosinophilia) and non-T2 inflammatory phenotypes (lack of atopy and eosinophilia, presence of neutrophilic and paucigranulocytic inflammation) is warranted.

Part of AZMs exacerbation sparing effect may be attributable to potential effects on the airway microbiome (20, 21). Findings from the AMAZES study for example suggest a greater exacerbation-sparing effect of AZM in the bacteria-positive group (18). In addition, antiviral properties have been described for AZM, with studies showing that AZM can augment RV-evoked IFN-β production in BECs from patients with asthma and COPD *in vitro* (22–25). However, whether AZMs antiviral properties may differ between patients with different inflammatory asthma phenotypes, thus explaining the lack of consensus on its clinical efficacy in different asthma populations, has not been investigated.

This study thus aimed at exploring bronchial epithelial antiviral properties in response to AZM in eosinophilic and non-eosinophilic as well as atopic and non-atopic asthma phenotypes. In addition, we investigated the effects of AZM on the release of RV-induced alarmins and pro-inflammatory cytokines in these different asthma phenotypes.

## Methods

### Patient characteristics

The present study had a cross-sectional design, and was conducted at Bispebjerg Hospital, Copenhagen, Denmark. Twenty patients with moderate-to-severe asthma were included. All patients had a confirmed diagnosis of asthma in accordance with the Global Initiative for Asthma (GINA) guidelines and were on maintenance treatment with inhaled corticosteroids (ICS) along with at least one second controller medication. Patients were non-smokers, defined as smoking history of less than 10 pack-years, and had experienced at least one systemic steroid-treated asthma exacerbation within the past year. A description of the timeline, visits, and all inclusion and exclusion criteria are available in the Supplementary Material.

Acute asthma exacerbation was defined as episodes of progressive worsening of symptoms from a stable state with an increase in shortness of breath, cough, wheezing, or chest tightness, resulting in increased use of asthma medication, visits to the emergency department, or hospitalization (26).

Patients were further stratified into subgroups based on peripheral blood eosinophil counts and atopic sensitization status. Eosinophilia was defined based on blood eosinophil count ≥0.2 × 10^9^/L, and non-eosinophilia was defined as blood eosinophil count <0.2 × 10^9^/L) (19). Atopy was defined sensitization to at least one aeroallergen, confirmed by both elevated specific IgE and a positive skin prick test. The standard aeroallergen panel included birch ([Betula species], grass [Phleum pratense] mugwort, horse, dog, cat [Felis domesticus], house dust mite [Der p 1 and Der f 2], and fungi [Alternaria and Cladosporium species].

### Human bronchial epithelial cell culture and stimulation

Patients underwent bronchoscopy (Olympus BF-1TQ180/BF-1TH190, Olympus, Hamburg, Germany) according to international guidelines (27). The complete procedure is described in the Supplementary Material.

BECs were cultured in bronchial epithelial growth medium (BEGM; Lonza, Switzerland) supplemented with 0.2% Primocin (InVivoGen, USA) and cultured at 37 °C and 5% CO2 in air. BECs were used at passage 2 and seeded into collagen coated 12-well plates in BEGM medium and were grown to 70%–80% confluence. BECs were then pre-treated with 10 µM AZM (Sigma-Aldrich, Denmark) for 24 h. The dose of AZM has been established for *in vitro* studies previously by this group (22, 23). Then BECs were infected with 0.05 MOI RV1B. RV1B was added to the cells for 1 h at room temperature with shaking. The virus was removed, and the cells were washed with phosphate-buffered saline (PBS). Fresh BEGM containing 10 µM AZM (Sigma-Aldrich, Denmark) was then added. Forty-eight hours post infection cell supernatants were collected for viral infectivity assay, measuring the 50% tissue culture infectious dose (TCID_50_) and protein release analyses. This time-point was again chosen based on previous studies, as we have shown that there is a robust induction of IFN-β protein release at 48 h post infection, which was not observed at 24 h (23).

### Viral progeny determination by TCID50

Cell supernatants were 1:10 serial diluted in DMEM with GlutaMAX containing 2% FBS, 1% penicillin-streptomycin, 1% non-essential amino acids, and 1% sodium pyruvate (Life Technologies, USA) and added to Ohio HeLa cells (European Collection of Cell Cultures, UK) in duplicates in 96-well plates. The plates were rocked for 1 h at room temperature and incubated for 4 days at 37 °C and 5% CO2. The cell monolayer was then fixed and stained with crystal violet, and cytopathic effects were assessed by spectrophotometry. TCID50 was calculated using the Spearman-Kärber algorithm.

### Mesoscale discovery (S-plex and U-plex) analysis of protein release

Protein release in cell culture supernatants from BECs was analyzed using MSD assays (Mesoscale Discovery, Maryland, USA). The release of IFN-β was assessed using an individual S-plex Mesoscale assay with a median lower limit of detection (LLOD) (16.8 fg/ml). Additionally, IL-6 (0.60 pg/ml), IL-8 (0.25 pg/ml), were analyzed utilizing the U-PLEX Custom Biomarker Group 1 (human) Assays from Mesoscale Discovery and median LLOD of IFN-λ (0.94 pg/ml), IL-1β (0.06 pg/ml), IL-33 (0.15 pg/ml), and TSLP (0.02 pg/ml) were analyzed using the U-PLEX Immuno-Oncology Group 1 (human assay). All protocols were performed according to the manufacturer's instructions. The data were acquired using a calibrated and validated instrument.

### Statistical analyses

The Mann–Whitney *U* test was used to compare two independent groups, while comparisons within groups were performed using the Wilcoxon signed-rank test. For comparisons of RV-infected vs. uninfected cells, multiple Wilcoxon tests with the Benjamini, Krieger, and Yekutieli method to control the false discovery rate (FDR) were performed for all cytokines in the total population. Similarly, comparing the log2FC of RV-infected vs. uninfected cells in the eosinophilic vs. non-eosinophilic groups, multiple Mann–Whitney *U* test with correction for multiple testing using the Benjamini, Krieger, and Yekutieli method (28) to control the false discovery rate (FDR) were performed.

The chi-squared test was used for categorical data. The Kolmogorov–Smirnov and Shapiro–Wilk tests were employed to assess the normality of the data distribution. No assumptions were made about missing data. Statistical significance was set at *p* < 0.05. Data analysis was performed using IBM SPSS Statistics version 29.01.0 and GraphPad Prism version 10.0.

### Power calculation

The power calculation for IFN-β and IFN-λ expression as primary read-out was based on previous *in vitro* studies with AZM (22, 23). A fold change of IFN-β was assumed to be at least 1.55 with a standard deviation of 0.555. With 80% power, a two-sided *α* level of 0.05, and accounting for a 20% dropout rate, a minimum of 8 patients per group were required.

## Results

### Clinical and demographic characteristics of the study population

Patients in the eosinophilic group exhibited significantly lower FEV1 and higher FeNO and total IgE levels than those in the non-eosinophilic group. The two groups were otherwise comparable (Supplementary Table S1). The patient characteristics of atopic and non-atopic asthma phenotypes are presented in Supplementary Table S2 and were likewise comparable between groups.

### RV induces the release of type I and III IFNs, alarmins, and pro-inflammatory cytokines across asthma phenotypes

**Figure 1:**
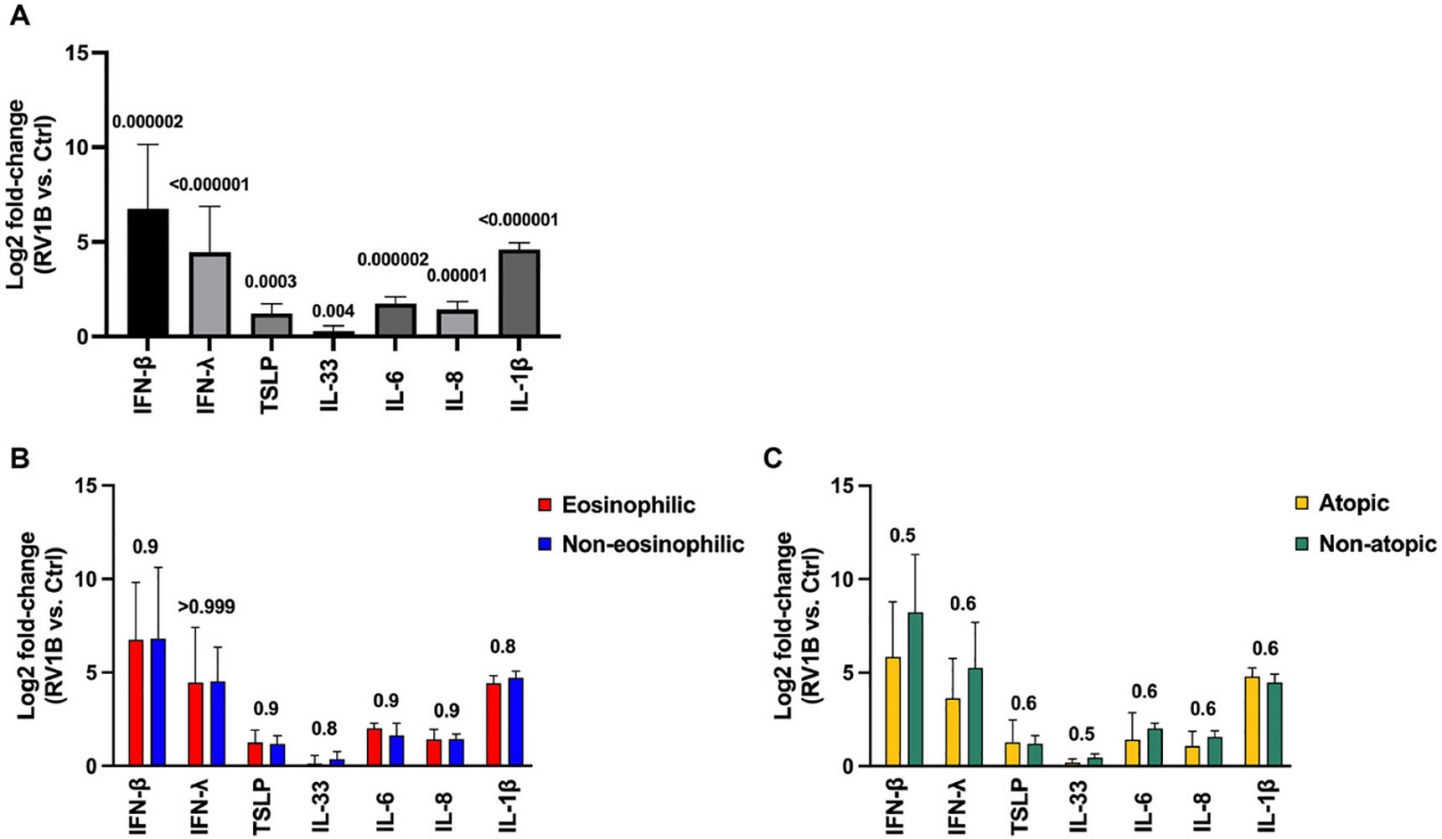
RV induces IFN-β, IFN-λ, alarmins, and pro-inflammatory cytokines in bronchial epithelial cells (BECs) from patients with moderate-to-severe asthma regardless of eosinophilia and atopy status. BECs were infected with 0.05 MOI RV1B for 48 h and cytokine release was analyzed in supernatants. **(A)** Log2 fold-change comparison of RV-induced cytokine release (compared with uninfected cells) from BECs in the **(A)** total population, **(B)** eosinophilic and non-eosinophilic subgroups, and **(C)** atopic and non-atopic subgroups. Cytokine protein levels were measured using MSD S-plexes and U-plexes. Statistical comparisons between and within groups were performed using multiple Mann–Whitney and Wilcoxon tests, respectively, with correction for multiple testing using the Benjamini, Krieger, and Yekutieli method to control the false discovery rate (FDR). Statistical significance was set at *p* < 0.05. *N* = 20 (10 for each phenotype).

Prior to assessing the effect of AZM, we established that *in vitro* RV infection induced a robust antiviral (IFN-β, IFN-λ) and inflammatory (TSLP, IL-33, IL-6, IL-8, IL-1β) response in BECs from our cohort (Figure 1A). Importantly, all the cytokine responses (to RV infection alone) were similar in the eosinophilic and non-eosinophilic phenotype (Figure 1B), and the atopic and non-atopic phenotype (Figure 1C).

### AZM augments BECs release of type I and III IFNs in patients with non-atopic asthma and regardless of eosinophilia

**Figure 2:**
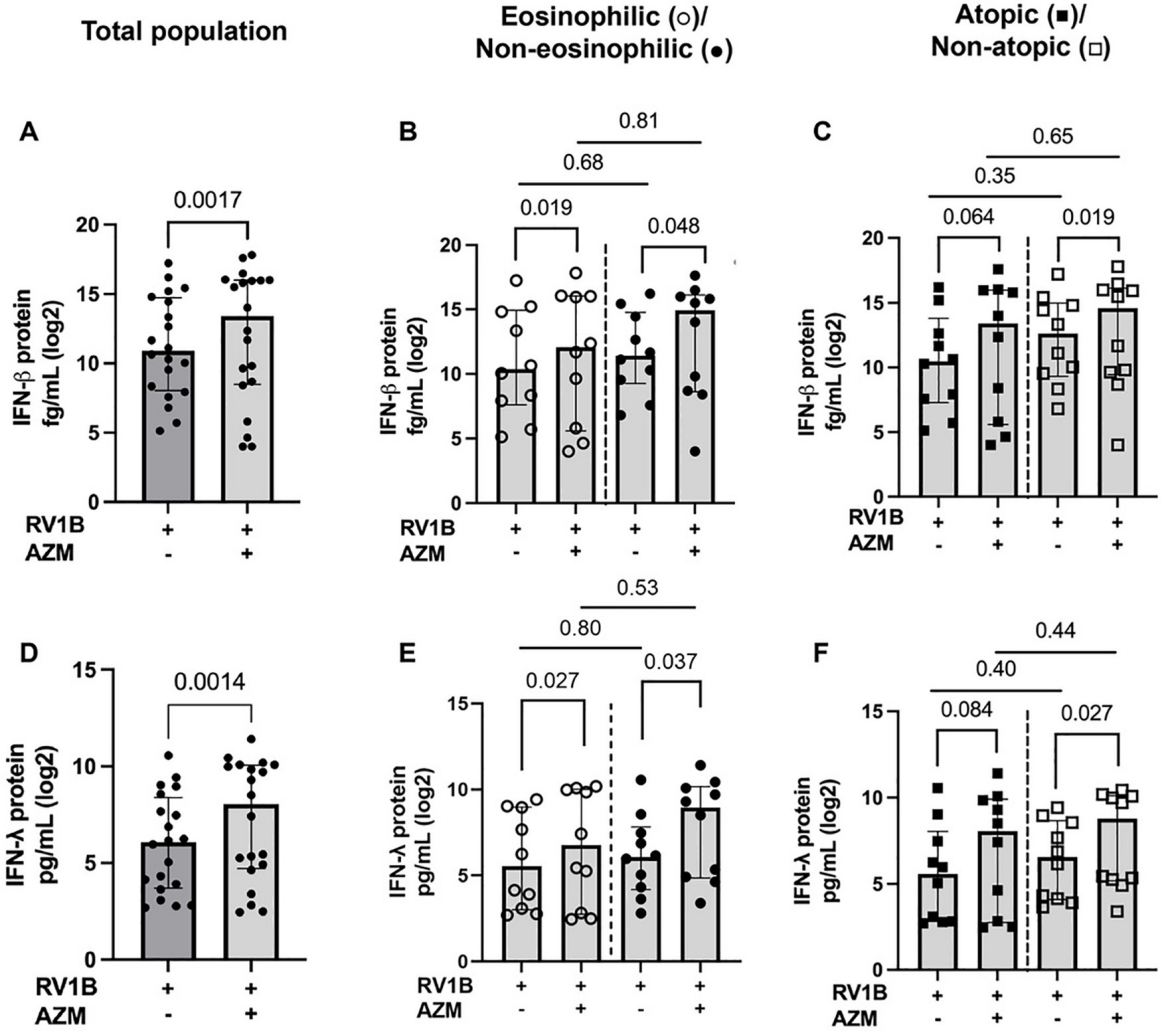
AZM significantly augments IFN-β and IFN-λ levels in eosinophilic, non-eosinophilic and non-atopic BECs from patients with moderate-to-severe asthma after RV infection. BECs were treated with AZM for 24 h before infection with 0.05 MOI RV. Release of IFN-β and IFN-λ were measured after 48 h of infection using the MSD S-plex and U-plex. **(A)** Log2 of absolute IFN-β protein release in the total population. **(B)** Log2 protein release of IFN-β in eosinophilic and non-eosinophilic phenotypes and **(C)** atopic and non-atopic phenotypes. **(D)** Log2 of absolute IFN-λ protein release in the total population. **(E)** Log2 protein release of IFN-λ in eosinophilic and non-eosinophilic phenotypes and **(F)** atopic and non-atopic phenotypes. Within-group comparisons were performed using the Wilcoxon Signed-rank test and between-group comparisons were made using the Mann–Whitney *U* test. Statistical significance was set at *p* < 0.05. *N* = 20 (10 for each phenotype).

Next, we confirmed that AZM improved the antiviral immunity in our cohort. Compared to untreated BECs infected with only RV, IFN-β and IFN-λ protein release was significantly upregulated following AZM treatment in the total population (*p* = 0.0017 and *p* = 0.0014, respectively) (Figures 2A,D). To determine whether the response to AZM differed between different inflammatory phenotypes of asthma, we then divided the patients into eosinophilic and non-eosinophilic, or atopic and non-atopic groups (as described in the methods section). AZM treatment increased the IFN-β and IFN-λ levels in both the eosinophilic (*p* = 0.019 and 0.027, respectively) and non-eosinophilic asthma phenotypes (*p* = 0.048 and 0.037, respectively) (Figures 2B,E). On the other hand, AZM only significantly increased the expression of IFN-β and λ in the non-atopic group (*p* = 0.019 and 0.027, respectively) (Figures 2C,F). In the atopic group, there was a notable trend towards an induction of IFN-β and IFN-λ, although this difference was not statistically significant (*p* = 0.064 and *p* = 0.084, respectively) (Figures 2C,F). Correcting for the difference in baseline lung function between groups did not affect this result.

### AZM treatment reduces viral infectivity (TCID50) in patients with non-atopic asthma and regardless of eosinophilia

**Figure 3:**
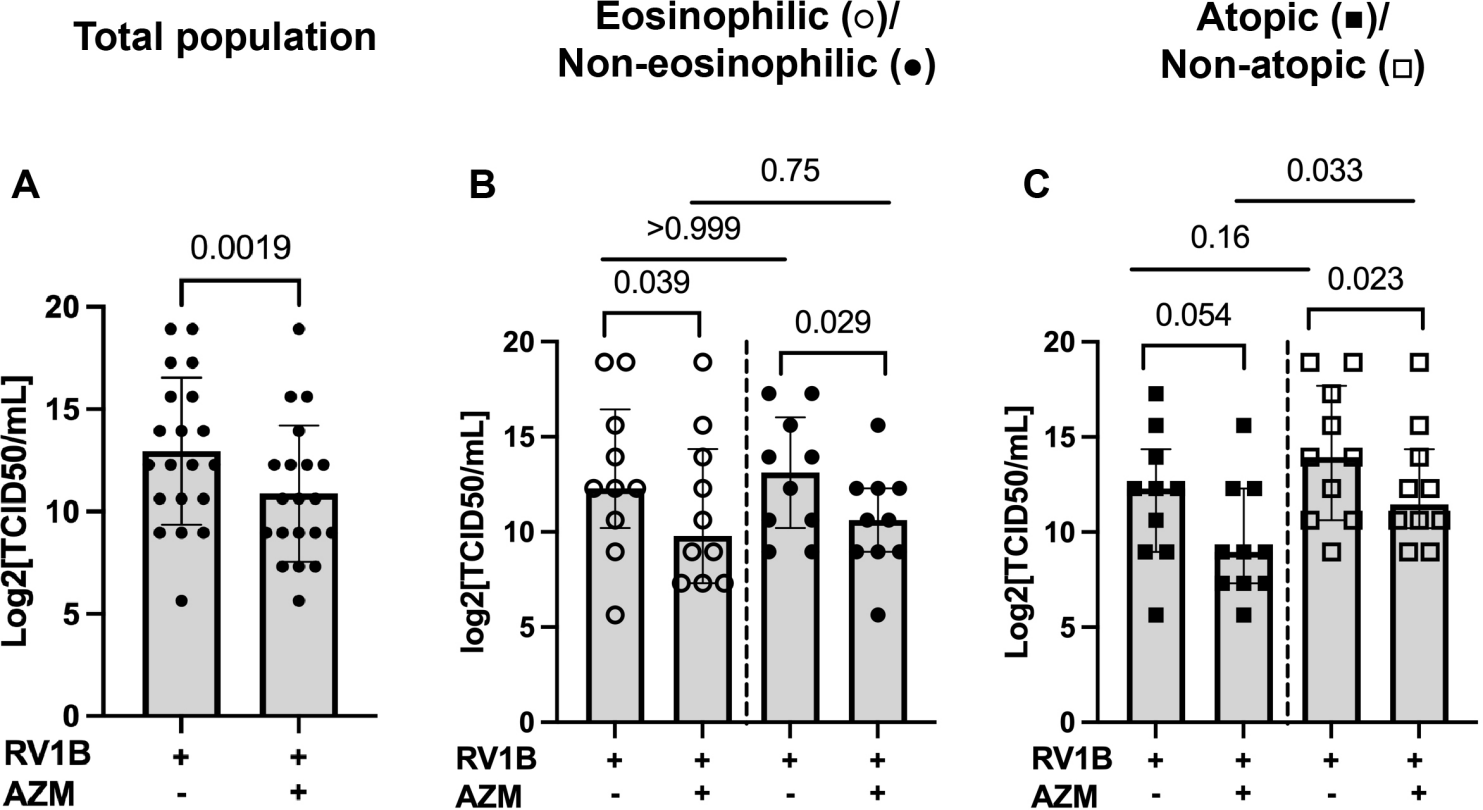
AZM inhibits viral infectivity in RV-infected BECs from moderate-to-severe asthma patients. BECs were treated with AZM for 24 h before infection with 0.05 MOI RV1B. Viral progeny in cell supernatants was measured 48 h after infection by TCID50 assay. **(A)** Log2 value of TCID50/ml in the total population. **(B)** Log2 TCID50/ml divided into eosinophilic and non-eosinophilic phenotypes. **(C)** Log2 TCID50/ml divided into atopic and non-atopic phenotypes. Within-group comparisons were performed using the Wilcoxon Signed-rank test and between-group comparisons were made using the Mann–Whitney *U* test. Statistical significance was set at *p* < 0.05. *N* = 20 (10 for each phenotype).

Compared with untreated RV-infected BECs, AZM also significantly reduced viral progeny in the total patient population (*p* = 0.0019), as well as in the eosinophilic and non-eosinophilic phenotypes (*p* = 0.039 and 0.029, respectively) (Figures 3A,B). In line with the IFN data, viral progeny was reduced by AZM in the non-atopic group (*p* = 0.023), but there was also an observed trend towards a reduction in viral progeny in the atopic phenotype (*p* = 0.054) (Figure 3C).

### AZM increases the release of RV-induced TSLP in BECs from a non-eosinophilic asthma phenotype only

**Figure 4:**
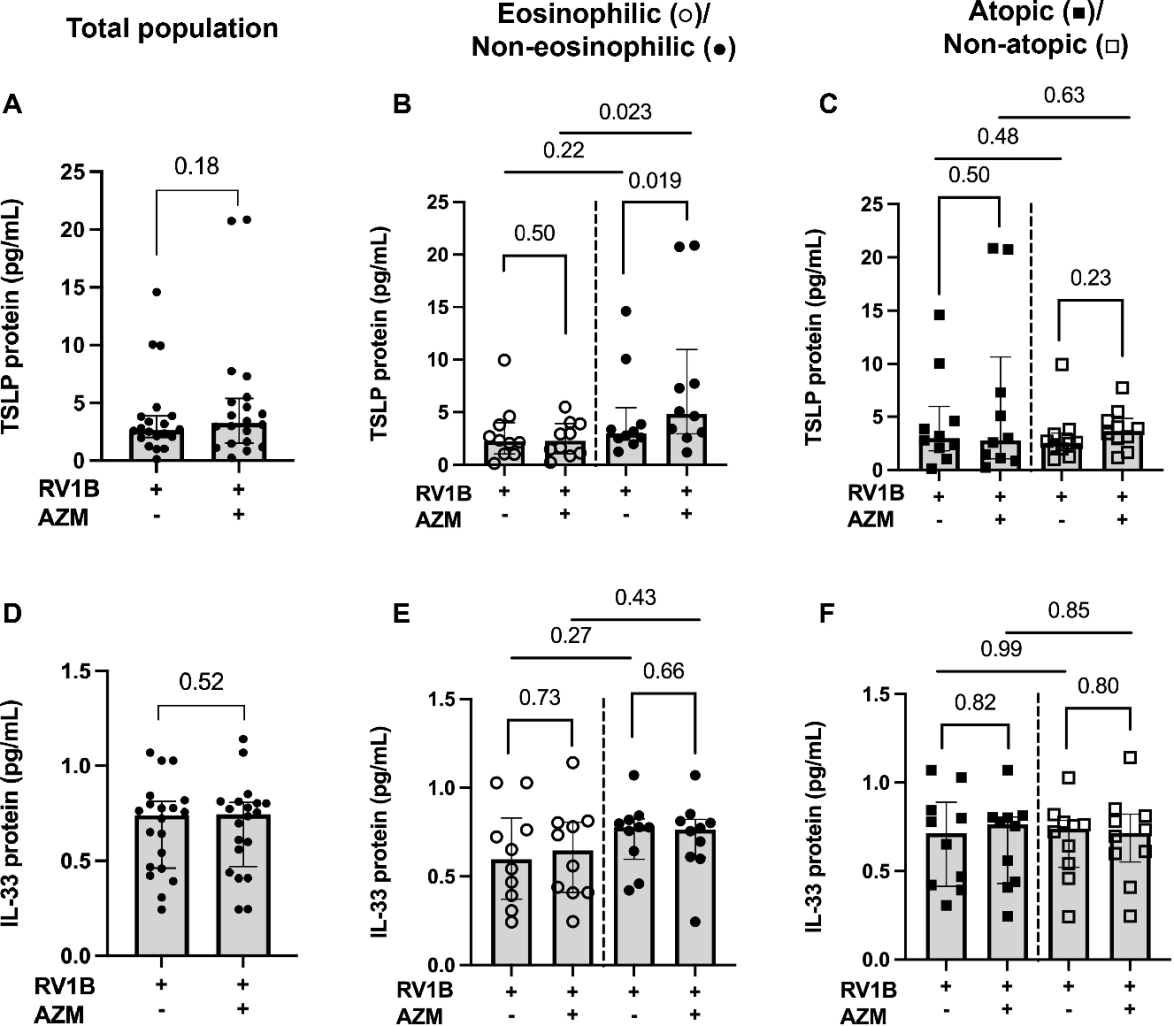
Effect of AZM on alarmin release in BECs from eosinophilic, non-eosinophilic, atopic and non-atopic asthma phenotypes. BECs were treated with AZM for 24 h before infection with 0.05 MOI RV1B, and protein release of TSLP and IL-33 was measured using MSD S-plex and U-plex 48 h after infection. **(A)** TSLP protein release from the total population. **(B)** Protein release of TSLP divided into eosinophilic and non-eosinophilic phenotypes and **(C)** atopic and non-atopic phenotypes. **(D)** IL-33 protein release from the total population. **(E)** Protein release of IL-33 divided into eosinophilic and non-eosinophilic phenotypes and **(F)** atopic and non-atopic phenotypes. Within-group comparisons were performed using the Wilcoxon Signed-rank test and between-group comparisons were made using the Mann–Whitney *U* test. Statistical significance was set at *p* < 0.05. *N* = 20 (10 for each phenotype).

AZM treatment did not significantly alter the RV-induced release of TSLP or IL-33 in the total patient population (*p* = 0.176 and *p* = 0.521, respectively) (Figures 4A,D). However, AZM treatment increased the RV-induced TSLP release in the non-eosinophilic phenotype (*p* = 0.019) (Figure 4B). Additionally, the release of TSLP in response to AZM was greater in the non-eosinophilic compared to the eosinophilic asthma phenotype (*p* = 0.023) (Figure 4B). In contrast, AZM did not affect the RV-induced IL-33 release in the eosinophilic or the non-eosinophilic phenotype (Figure 4E). There were no significant changes in the expression of either TSLP nor IL-33 in the atopic or non-atopic groups (Figures 4C,F).

### AZM has a limited effect on RV-induced release of pro-inflammatory cytokines in BECs from different inflammatory asthma phenotypes

**Figure 5:**
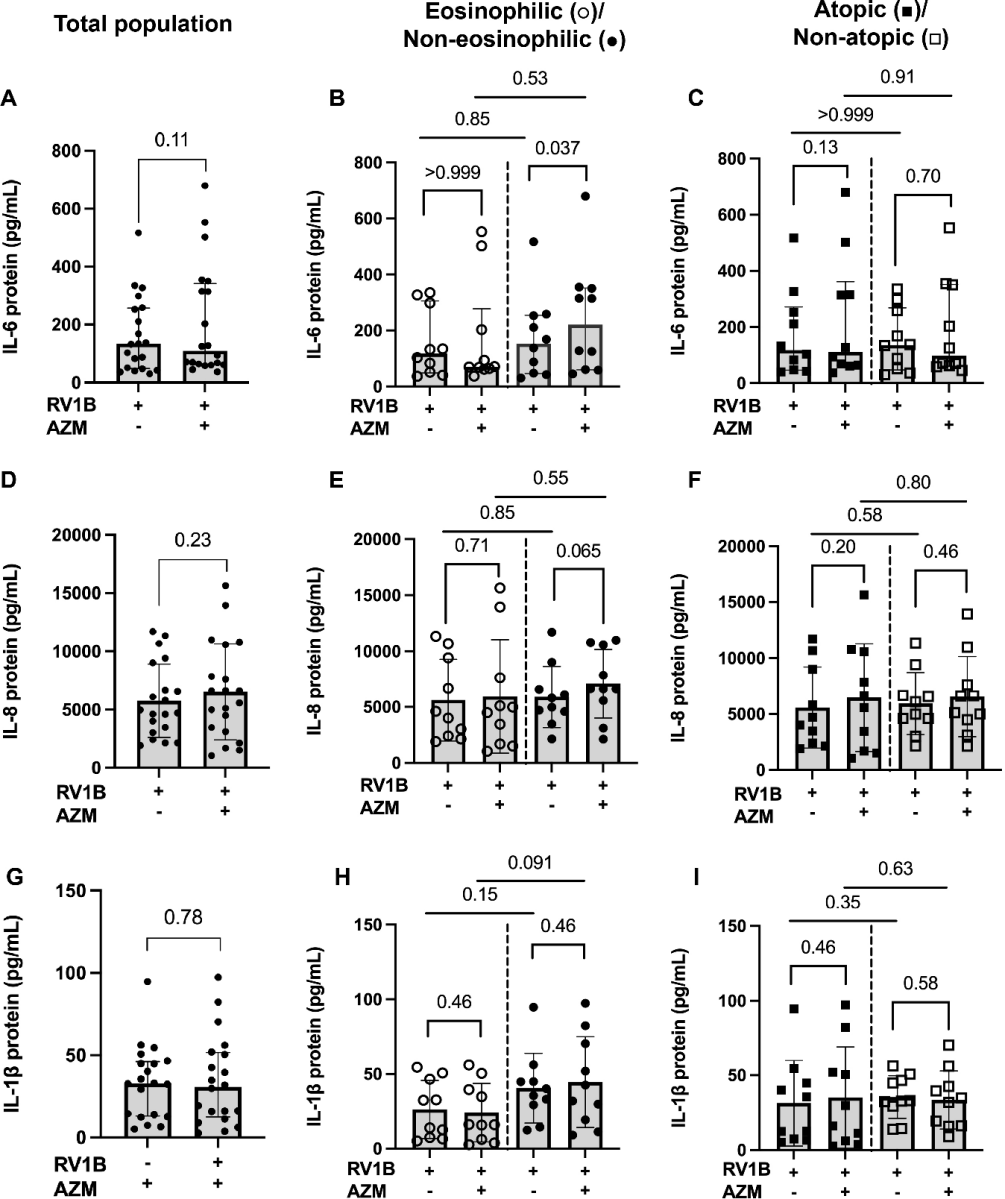
Effect of AZM on pro-inflammatory release in BECs from eosinophilic, non-eosinophilic, atopic and non-atopic asthma phenotypes. BECs were treated with AZM for 24 h before infection with 0.05 MOI RV1B, and protein release of IL-6, IL-8 and IL-1β was measured using MSD S-plex and U-plex 48 h after infection. **(A)** Release of IL-6 protein in the total population. **(B)** Release of IL-6 in eosinophilic and non-eosinophilic and **(C)** atopic and non-atopic phenotypes. **(D)** Release of IL-8 protein in the total population. **(E)** Release of IL-8 in eosinophilic and non-eosinophilic and **(F)** atopic and non-atopic phenotypes. **(G)** Release of IL-1β protein in the total population. **(H)** Release of IL-1β in eosinophilic and non-eosinophilic and **(I)** atopic and non-atopic phenotypes. Within-group comparisons were performed using the Wilcoxon Signed-rank test and between-group comparisons were made using the Mann–Whitney *U* test. Statistical significance was set at *p* < 0.05. *N* = 20 (10 for each phenotype).

AZM treatment did not alter the RV-induced release of IL-6, IL-8 or IL-1β in the total patient population (Figures 5A,D,G). AZM treatment resulted in an increased release of IL-6 (*p* = 0.037) as well as a trend towards an increase in IL-8 release (*p* = 0.065) in response to RV in the non-eosinophilic asthma phenotype (Figures 5B,E). When the eosinophilic and non-eosinophilic phenotypes were compared, no significant differences were observed in the AZM-induced release of IL-6 and IL-8 (Figures 5B,E). AZM treatment did not alter the RV-induced IL-1β release in either the eosinophilic or non-eosinophilic asthma phenotype (Figure 5H). Furthermore, AZM treatment had no effect on the RV-induced release of IL-6, IL-8 or IL-1β in neither the atopic nor non-atopic phenotype (Figures 5C,F,I).

## Discussion

In this study, we provide *in vitro* evidence that the antiviral effects of AZM are generally similar in BECs from moderate-to-severe asthma patients with different inflammatory phenotypes. AZM treatment increased RV-induced IFN-β and IFN-λ protein release and reduced viral progeny in patients with non-atopic asthma, and a similar trend was observed in atopic asthma, and regardless of eosinophilia. This suggests that AZM enhances epithelial resistance to viral infections across asthma phenotypes, and that its antiviral actions are independent of T2 immune pathways. These findings advance our understanding of AZM's exacerbation-sparing effects in moderate-to-severe asthma and underscore the broader implications of AZM for managing asthma exacerbations.

This study is the first to examine the differential effects of AZM on BEC responses to RV infection in moderate-to-severe asthma, differentiating between eosinophilic and non-eosinophilic, as well as atopic and non-atopic phenotypes. Our findings show that AZM enhances the release of type I and type III IFNs in infected BECs, which aligns with previous studies by us and others indicating that macrolide antibiotics can boost antiviral immune responses in BECs (22–25). Notably, AZM augmented RV-induced IFNs largely independent of asthma inflammatory phenotype, suggesting a broad antiviral potential. Additionally, AZM significantly reduced viral progeny independent of inflammatory phenotype and atopy status, which is consistent with the increased IFN response, highlighting the potential of AZM to mitigate viral exacerbations in various asthma phenotypes. This is particularly relevant given the high morbidity associated with viral infections in asthma.

Given these findings, it remains important to clarify which asthma phenotypes benefit most from AZM treatment. While AMAZES (18) reported that AZM reduced moderate-to-severe asthma exacerbations in both eosinophilic and non-eosinophilic phenotypes, the AZISAST study (19) reported that AZM decreased severe asthma exacerbations especially in the non-eosinophilic asthma phenotype. There are notable similarities and differences in study populations and inclusion criteria between these studies and ours that may explain discrepancies in these observations. While AMAZES (18) defined the inflammatory phenotypes by either a sputum eosinophil count of at least 3% or a blood eosinophil count over 300/µl, in our study non-eosinophilic asthma was defined by blood eosinophil counts below 200/µl, which is like the AZISAST study, and might better represent true non-eosinophilic asthma. Differences in our findings to those in AZISAST may, however, be attributable to differences in inclusion criteria as AZISAST required patients to have had at least two severe asthma exacerbations, while our study only required one exacerbation. In addition, although these previous studies included mostly patients with allergic asthma, the impact of atopy status on AZM response was not reported. Our findings suggest that the improved antiviral response *in vitro* is similar in atopic and non-atopic patients, which suggests a clinical benefit of AZM in both atopic and non-atopic patients.

The dose of AZM (10 µM) was determined according to previous studies performed by our research group (22, 23). In these studies, doses of 0.4–25 µM were chosen based on a study by Di Paolo et al. (29), where clinical administration of 500 mg AZM led to maximum concentrations in lung tissue and bronchial washings of approximately 10 and 1 µM. In both of our previous works, maximum induction of IFN-β was achieved by 10 µM AZM, and hence, this dose was chosen for the present study.

Our study confirmed that RV infection triggers TSLP and IL-33 release across the entire population, which is consistent with previous research (5, 30–31). However, AZM treatment did not affect the release of these alarmins across different inflammatory asthma phenotypes. Notably, AZM treatment increased TSLP release only in non-eosinophilic asthma patients, a novel finding that warrants further investigation in larger studies. While previous research has shown that AZM inhibits TSLP release (32), we speculate that the discrepancy may be due to differences in experimental setups. In the aforementioned study, TSLP was measured at gene level in normal BECs exposed to poly (I:C) in a T2 cytokine environment, a set up that differs remarkably to ours. Additionally, the differential responses may result from intrinsic differences between normal and asthmatic BECs, including variations in proliferation rates, cytokine secretion, and injury susceptibility (10, 33–34). Furthermore, AZM had no significant effect on IL-33 release in our study using human bronchial epithelial cells from asthma patients. In contrast, earlier studies reported that AZM reduced IL-33 gene expression in bronchoalveolar lavage (BAL) fluid from an unstimulated mouse model of asthma, where multiple cell types might contribute to IL-33 release, potentially explaining the conflicting results (35).

Pro-inflammatory cytokines (IL-6, IL-8, and IL-1β) were induced following RV infection in all patients but remained primarily unchanged with AZM treatment, consistent with prior studies (22, 25). These findings indicate that the primary antiviral effect of AZM may not involve modulating pro-inflammatory pathways. However, our study noted increased IL-6 induction and a trend towards increased IL-8 in BECs from non-eosinophilic patients treated with AZM. This suggests a modulation of specific inflammatory pathways in non-eosinophilic asthma, which often poorly responds to standard steroid therapies. IL-6, a critical cytokine in immune regulation and inflammation, may enhance host defense against viral infections in non-eosinophilic asthma (36). On the other hand, IL-6 has been associated with a decrease in lung function in severe asthma (37) and may induce mucus hypersecretion (38). Systemic IL-6 inflammation has been further linked to metabolic dysfunction and asthma severity (39). Similarly, IL-8, a key mediator of inflammation, functions primarily as a neutrophil chemoattractant. The observed increase in IL-8 may indicate a heightened ability to recruit neutrophils, potentially aiding in the clearance of viral and bacterial pathogens (40), but it may also exacerbate inflammation. Further research is necessary to elucidate the clinical implications of these *in vitro* findings, which is essential for tailoring treatments for non-eosinophilic asthma and optimizing AZM as an adjunct therapy in asthma management.

While this study provides novel insights into the antiviral effects of AZM across asthma phenotypes, certain methodological considerations should be noted. The relatively small sample size may reduce the ability to detect more modest differences between subgroups and limits broader generalizability. However, using a paired *in vitro* design where each patient served as their own control helped strengthen the internal validity and allowed for meaningful within-subject comparisons. Classification into eosinophilic or non-eosinophilic and atopic or non-atopic subgroups was based on standard clinical definitions. Importantly, the distribution of atopic and non-atopic patients was balanced within each eosinophil-defined group, with an equal number of patients in each category. This balanced design reduces the risk of confounding due to overlapping phenotypes and supports the interpretation of observed differences in total IgE. In addition, although all patients met criteria for moderate-to-severe asthma according to GINA guidelines, the study was not specifically powered to assess differences in response based on severity level. Lastly, although bronchoscopy was essential for obtaining BECs, its invasive nature may have influenced which patients agreed to participate in the study.

Although *in vivo* studies would provide important complementary evidence, direct clinical administration of AZM followed by RV challenge would pose ethical and safety concerns, especially in patients with severe asthma. Thus, our *in vitro* approach can also be regarded as a strength, as previous studies indicate that the inflammatory phenotype of BECs are preserved *in vitro* (12), providing an environment that closely mimics *in vivo* conditions without patient risk. Additionally, this method allowed us to examine the impact of AZM on viral progeny. Submerged BEC culture is a well-established model for replicating innate immune responses and is widely accepted for inducing relevant pro-inflammatory and antiviral mediators in bronchial epithelial cells (11, 41). Since our study did not focus on epithelial barrier function, airway mucus production, remodeling, or other asthma-related features, the use of air-liquid interface cultures were not considered essential for our objectives.

This study contributes to an enhanced understanding of the molecular actions of AZM's exacerbation-sparing effects in patients with asthma with a history of exacerbation, independent of T2 phenotypes. Elevated levels of antiviral cytokines after AZM treatment may be correlated with a stronger immune response to viral infections, potentially leading to fewer exacerbations. The implications of our findings are significant, suggesting that AZM treatment could be particularly beneficial in enhancing viral resistance across a diverse set of inflammatory asthma phenotypes without exacerbating inflammatory cytokine responses. Furthermore, its efficacy in reducing viral progeny makes AZM interesting. However, future studies should focus on *in vivo* clinical treatment with AZM to better understand how these *in vitro* findings translate into clinical efficacy, particularly in reducing asthma exacerbations and improving the quality of life of patients with asthma. In addition, it is important to identify predictors of response to Azithromycin, which are currently lacking, to guide clinicians in step 5 GINA management decisions. Our findings suggest that AZM treatment should not be guided by T2 or atopy status, which contrasts treatment initiation with biologicals. Studies focusing on biomarkers for viral induced exacerbations or impaired antiviral responses could therefore aid more targeted Azithromycin treatment.

## Conclusions

In conclusion, our data suggest that AZM improves the antiviral resistance of BECs by enhancing type I and III interferon production, independent of blood eosinophil counts and atopy status, and further support the clinical evidence that AZM is effective in reducing asthma exacerbations independent of inflammatory phenotype.

## Data Availability

The raw data supporting the conclusions of this article will be made available by the authors, without undue reservation.
