# Supplementary material for: Effects of in vitro azithromycin treatment on bronchial epithelial antiviral immunity in asthma phenotypes
Source: Front Allergy. 2025 Jun 17;6:1605109. doi: 10.3389/falgy.2025.1605109 (PMC12209221; doi:10.3389/falgy.2025.1605109)
Supplement: Supplementary file 1 [file Datasheet1.docx]

# Effects of *in vitro* azithromycin treatment on bronchial epithelial antiviral immunity in asthma phenotypes

**Author list**

Muzhda Ghanizada^1^, Sofia Malm Tillgren^2^, Louis Praeger-Jahnsen^3^, Nihaya Mahmoud Said^1^, Sisse Ditlev^3^, Helle Frost Andreassen^1^, Nanna Dyhre-Petersen^1^, Samuel Cerps^2^, Asger Sverrild^1^, Celeste Porsbjerg^1^, Lena Uller^2^, Therese Lapperre^1,4,5^ Mandy Menzel^2^.

M. Ghanizada and S.M. Tillgren contributed to manuscript equally.

**Affiliations**

1: Respiratory Research Unit, Department of Respiratory and Infectious Diseases, Bispebjerg Hospital, Copenhagen, Denmark.

2: Unit of Respiratory Immunopharmacology, Department of Experimental Medical Science, Lund University, Sweden

3: Copenhagen Centre for Translational Research, Copenhagen University Hospital Bispebjerg and Frederiksberg, Bispebjerg Bakke 23, 2400 Copenhagen, Denmark.

4: Department of Respiratory Medicine University Hospital, Antwerp Edegem, Belgium.

5: Laboratory of Experimental Medicine and Paediatrics University of Antwerp Wilrijk, Belgium

**Study plan and timing**

This is a cross-sectional study conducted at Bispebjerg Hospital, Copenhagen, Denmark, which was approved by the Danish ethics committee (H-19067267).

**Visit 1 (baseline):** asthma control questionnaire (ACQ-6), mini Asthma Quality of Life Questionnaire (MiniAQLQ), forced expiratory volume in 1 s (FEV1), fractioned exhaled nitric oxide (FeNO), airway hyperresponsiveness (AHR) to mannitol, reversibility to β2-agonist, Electrocardiogram (ECG), skin prick test, blood test included but not limited to blood leukocytes, blood eosinophils counts, total IgE, radioallergosorbent test (RAST) for 10 standard aeroallergens, and High-resolution computed tomography (HRCT) was performed in order to exclude significant other chronic lung diseases such as bronchiectasis or interstitial lung disease or cancer.

**Visit 2:** Patients underwent bronchoscopy with taking bronchial brushes to sample bronchial epithelial cells.

**Full list of inclusion and exclusion criteria**

Subjects met *all* the following inclusion criteria:

1. Signed informed consent.
2. Age ≥18 through 75 years
3. Diagnosis of asthma according to GINA, with confirmed variable airflow obstruction at screening visit or previously Reversibility to ß^2^-agonist
4. A postbronchodilator FEV_1_ ≥ 50% predicted.
5. Maintenance treatment with ICS and ≥1 second controller (LABA, LAMA, LTRA or Xanthines) for at least three months prior to V1
6. Non-smokers (<10 packyears, quit >6 months).
7. ≥1 Systemic steroid treated exacerbation in the past one year despite maintenance treatment with inhaled corticosteroids.
8. Negative urin-hcg test for female subjects of childbearing potential at V1
9. Females of childbearing potential who are sexually active with a non-sterilized male partner must use a highly effective method of contraception from the time informed consent is obtained and must agree to continue using such precautions through Week 12 of the study; cessation of contraception after this point should be discussed with a responsible physician. Periodic abstinence, the rhythm method, and the withdrawal method are not acceptable methods of contraception. Females of childbearing potential are defined as those who are not surgically sterile (ie, bilateral tubal ligation, bilateral oophorectomy, or complete hysterectomy) or postmenopausal (defined as 12 months with no menses without an alternative medical cause).
10. Fertile males must use condom.

Any of the following would exclude the subject from participation in the study:

1. Previous medical history or evidence of an uncontrolled intercurrent illness that in the opinion of the investigator may compromise the safety of the subject in the study or interfere with evaluation of the investigational product or reduce the subject’s ability to participate in the study. Subjects with well-controlled comorbid disease (e.g., hypertension, hyperlipidaemia, gastroesophageal reflux disease) on a stable treatment regimen for 15 days prior to Visit 1 are eligible.
2. Any concomitant respiratory disease that in the opinion of the investigator will interfere with the evaluation of the investigational product or interpretation of subject safety or study results (e.g., cystic fibrosis, pulmonary fibrosis, moderate-severe bronchiectasis, allergic bronchopulmonary aspergillosis, Churg-Strauss syndrome, active tuberculosis).
3. Patient with asthma: concomitant COPD.
4. Any clinically relevant abnormal findings in haematology or clinical chemistry (laboratory results from Visit 1), physical examination, vital signs during the screening, which in the opinion of the investigator, may put the subject at risk because of his/her participation in the study, or may influence the results of the study, or the subject’s ability to participate in the study.
5. Evidence of active liver disease, including jaundice or aspartate transaminase, alanine transaminase, or alkaline phosphatase >1.5 times the upper limit of normal (laboratory results from Visit 1).
6. GFR <30 ml/min.
7. Acute upper or lower respiratory infections requiring antibiotics or antiviral medications within 2 weeks prior to Visit 1 or at Visit 2 (randomization).
8. A positive human immunodeficiency virus (HIV) test before study or subject taking antiretroviral medications, as determined by medical history and/or subject’s verbal report.
9. History of sensitivity to any component of the investigational product formulation or a history of drug or other allergy that, in the opinion of the investigator contraindicates their participation.
10. History of any known primary immunodeficiency disorder excluding asymptomatic selective immunoglobulin A or IgG subclass deficiency.

Receipt of any of the following within 30 days prior to Visit 1:

1. Immunoglobulin or blood products, or
2. Receipt of any investigational non-biologic agent within 30 days or 5 half-lives prior Visit 1, whichever is longer.
3. Pregnant, breastfeeding, or lactating females
4. History of chronic alcohol or drug abuse within 12 months prior to Visit 1.
5. Planned surgical procedures requiring general anaesthesia or in-patient status for > 1 day during the conduct of the study.
6. Unwillingness or inability to follow the procedures outlined in the protocol.
7. Concurrent enrolment in another clinical study involving an investigational treatment.
8. Receipt of any live or attenuated vaccines within 15 days prior to Visit 1.
9. Long QTc interval on ECG (QTc >480msec).

History of the following cardiac comorbidities:

1. Life-threatening arrhythmias
2. Myocardial infarction (NSTEMI or STEMI) less than 6 months before start of the study
3. Unstable angina
4. History of severe heart failure
5. Documented severe hypokalaemia (K <3.0 mmol/L) or hypomagnesemia (Mg <0.5 mmol/L).
6. Life expectancy <6 months.
7. Hearing impairment.
8. Oxygen saturation <92% at room air, patients on LTOT, history of chronic respiratory failure (hypercapnia).

Excluded medications

1. Drugs with a risk for long QTc interval and torsade de pointes (Table 1).
2. Drugs that interact with AZM in terms of risk of rhabdomyolysis, such as Simvastatin. Atorvastatin but Fluvastatin or Rosuvastatin may be prescribed instead.
3. Oral corticosteroids (any dose for more than 3 days) or maintenance macrolide treatment 12 weeks prior to Visit 1 or during the run-in period.
4. Use of immunosuppressive medication (e.g., methotrexate, troleandomycin, oral gold, cyclosporine, azathioprine, intramuscular long-acting depot corticosteroid, or any experimental anti-inflammatory therapy) within 3 months prior to Visit 1 and during the study.
5. Receipt of any oral or ophthalmic β-adrenergic antagonists (e.g., propranolol) within 15 days prior to Visit 1.
6. Use of anticoagulation treatment (warfarin, clopidogrel) that cannot be withheld prior to bronchoscopy.
7. Receipt of any marketed (including omalizumab) or investigational biologic agent within 4 months or 5 half-lives prior to Visit 1, whichever is longer.
8. Allergen immunotherapy
9. Digoxin and colchicine treatment during the study.
10. Antacids: not excluded, but may reduce plasma levels of AZM, and therefore need to be taken >1 hour before AZM or 2 hours after AZM is taken

Procedures

Bronchoscopy

BECs were obtained from the patients with bronchoscope (Olympus BF-1TQ180/BF-1TH190, Olympus, Hamburg, Germany) with standard sterile‐sheared nylon cytology brushes as previously described [1]. Bronchoscopy was performed according to international guidelines [2]. Bronchial brushings were obtained under local anaesthesia and sedation with midazolam and fentanyl. Bronchial brushings (Cytobrush, Ø3mm, 10mm, Olympus Medical Systems Corp., Japan) were performed in the left lower lobe (segment 8-10) in V2.

Statistical adjustment

To assess whether differences in lung function (FEV₁ or FVC) influenced cytokine responses to RV and AZM treatment, we conducted Spearman correlation analyses within eosinophilic and non-eosinophilic groups. We then performed linear regression analyses using IFN-β response as the dependent variable, asthma phenotypes as the independent variable, and FEV₁ % or FVC % as covariates. No significant associations or adjusted group differences were identified.

# **Table S1: Clinical and Demographic characteristics of eosinophilic and non-eosinophilic asthma phenotypes**

|  | Total  (n=20) | Eosinophilic  (n=10) | Non-eosinophilic  (n=10) | P-value |
| --- | --- | --- | --- | --- |
| **Demographic characteristics** | | | | |
| Age (years) | 39 (28-49) | 42 (30-61) | 36 (27-45) | 0.28 |
| Female, n (%) | 16 (80) | 6 (60) | 10 (100) | 0.08 |
| BMI (kg/m^2^) | 24.6 (23.6-31.0) | 28.6 (22.9-31.5) | 24.5 (23.7-29.1) | 0.58 |
| Ex-smoker, n (%) | 4 (20) | 2 (20) | 2 (20) | 1.00 |
| Pack years | 0 (0-0) | 0.0 (0.0-1.0) | 0.0 (0.0-0.62) | 0.97 |
| **Asthma severity** | | | | |
| Number of exacerbations in last 12 months | 1 (1-2.75) | 2 (1-3) | 1 (1-2.75) | 0.34 |
| ACQ_6_ | 2.3 (1.9-2.7) | 2.5 (1.8-3.4) | 2.2 (1.9-2.4) | 0.19 |
| **Lung function (pre-SABA)** | | | | |
| FEV_1_ (%) | 85.5 (65.5-98.5) | 73.5 (60-88) | 97 (80-103) | 0.02 |
| FVC (%) | 92 (83.3-98.5) | 84.5 (70.8-92) | 97 (91.5-101.8) | 0.01 |
| FEV_1_/FVC (%) | 75 (67-83) | 72 (65-80) | 80 (71-84) | 0.10 |
| PD15 mannitol, (n=14), mg | 635 (435-635) | 635 (315-635) | 635 (395-635) | 0.89 |
| **Medication** | | | | |
| ICS (budesonide equivalent) | 1600 (800-1600) | 1600 (1400-1600) | 1000 (800-1600) | 0.09 |
| ICS/LABA, n (%) | 19 (95) | 9 (90) | 10 (100) | 0.5 |
| LAMA, n (%) | 9 (45) | 6 (60) | 3 (30) | 0.18 |
| Montelukast, n (%) | 11 (55) | 6 (60) | 5 (50) | 0.5 |
| SABA as needed. Puff/day | 1.5 (0-14.5) | 1.5 (1.5-14.5) | 1.5 (0-3.5) | 0.08 |
| **T2 biomarkers** | | | | |
| FeNO, ppb | 16 (9-20) | 19 (15-49) | 10 (8-16) | 0.02 |
| Prick test positive, n, (%) | 10 (50) | 5 (50) | 5 (50) | 1.00 |
| Total IgE (kU_a_*L^-1^) | 52 (21-189) | 118 (52-868) | 30.5 (3.8-57) | 0.01 |
| Eosinophils (10^9^ L^-1^) | 0.15 (0.08-0.38) | 0.37 (0.25-0.79) | 0.085 (0.04-0.10) | <0.001 |
| **Non-T2 biomarker** | | | | |
| Blood Neutrophils (10^9^ L^-1^) | 4.1 (2.9-4.8) | 4.5 (3.8-5.5) | 3.3 (2.8-4.5) | 0.075 |

**Legend Table S1:** Eosinophilia is defined as a serum eosinophil ≥ 0.2 109 cells/L. Data are presented as medians with interquartile ranges, n (%). BMI: body mass index. ACQ_6_: Asthma Control Questionnaire – 6 items. Pack-years: packs of cigarettes smoked per day multiplied by the number of years smoked. FEV1, forced expiratory volume in 1 s. FVC: forced vital capacity. FeNO: Fractional exhaled nitric oxide. ICS, inhaled corticosteroids; ICS/LABA: Inhaled Corticosteroids/Long-Acting Beta2-Agonists. LAMA: Long-Acting Muscarinic Antagonist. SABA: Short-acting beta agonists. The chi-square test and Mann–Whitney U-test were used for non-parametric categorical and skewed continuous variables, respectively. Fisher’s Exact Test was used for expected counts < 5. Statistical significance was set at p < 0.05.

# **Table S****2: Clinical and Demographic characteristics of atopic and non-atopic asthma phenotypes**

|  | Total  (n=20) | Atopic  (n=10) | Non-atopic  (n=10) | P-value |
| --- | --- | --- | --- | --- |
| **Demographic characteristics** | | | | |
| Age (years) | 39 (28-49) | 37 (29-45) | 43 (27-52) | 0.38 |
| Female, n (%) | 16 (80) | 9 (90) | 7 (70) | 0.26 |
| BMI (kg/m^2^) | 24.6 (23.6-31.0) | 24.4 (22-31) | 27.9 (24-31) | 0.36 |
| Ex-smoker, n (%) | 4 (20) | 3 (30) | 1 (10) | 0.58 |
| Pack years | 0 (0.0-0.0) | 0 (0.0-4.3) | 0 (0.0-0.0) | 0.21 |
| **Asthma severity** | | | | |
| Number of exacerbations in last 12 months | 1 (1-2.75) | 1 (1-3.5) | 1.5 (1-2.25) | 0.86 |
| ACQ_6_ | 2.3 (1.9-2.7) | 2.1 (1.5-2.6) | 2.3 (2.1-3.4) | 0.28 |
| **Lung function (pre-SABA)** | | | | |
| FEV_1_ (%) | 85.5 (65.5-98.5) | 88.5 (64.7-101.2) | 84 (69.2-96.7) | 0.76 |
| FVC (%) | 92 (83.3-98.5) | 94 (74-104.7) | 89 (83.9-94.7) | 0.34 |
| FEV_1_/FVC (%) | 75 (67-83) | 73 (68-81) | 79 (63.4-83.6) | 0.94 |
| PD15 mannitol, n=14, mg | 635 (435-635) | 635 (515-635) | 555 (355-635) | 0.32 |
| **Medication** | | | | |
| ICS (budesonide equivalent) | 1600 (800-1600) | 800 (800-1600) | 800 (800-1600) | 0.45 |
| ICS/LABA, n (%) | 19 (95) | 9 (90) | 10 (100) | 1.0 |
| LAMA, n (%) | 9 (45) | 5 (50) | 4 (40) | 1.0 |
| Montelukast, n (%) | 11 (55) | 6 (60) | 5 (50) | 0.65 |
| SABA as needed. Puff/day | 1.5 (0-14.5) | 1.5 (1.5-3.5) | 1.5 (1.1-3.5) | 0.80 |
| **T2 biomarkers** | | | | |
| FeNO, ppb | 16 (9-20) | 15.2 (8.1-19) | 16.3 (9.3-51.8) | 0.36 |
| Total IgE (kU_a_*L^-1^) | 52 (21-189) | 137 (31.7-868) | 48.5 (3.75-70.0) | 0.07 |
| Eosinophils (10^9^ L^-1^) | 0.15 (0.08-0.38) | 0.21 (0.09-0.48) | 0.15 (0.05-0.35) | 0.52 |
| **Non-T2 biomarker** | | | | |
| Neutrophils (10^9^ L^-1^) | 4.1 (2.9-4.8) | 3.8 (2.8-4.9) | 4.3 (2.9-4.9) | 0.71 |

**Legend Table S2:** Atopy was defined with atopic sensitization with both elevated specific IgE and a positive skin prick test for at least one aeroallergen in a standard panel of ten aeroallergens: birch ([Betula species], grass [Phleum pratense] mugwort, horse, dog, cat [Felis domesticus], house dust mite [Der p 1 and Der f 2], and fungi [Alternaria and Cladosporium species]. Data are presented as medians with interquartile ranges, n (%). BMI: body mass index. ACQ_6_: Asthma Control Questionnaire – 6 items. Pack-years: packs of cigarettes smoked per day multiplied by the number of years smoked. FEV1, forced expiratory volume in 1 s. FVC: forced vital capacity. FeNO: Fractional exhaled nitric oxide. ICS: inhaled corticosteroids; ICS/LABA: Inhaled Corticosteroids/Long-Acting Beta2-Agonists. LAMA: Long-Acting Muscarinic Antagonist. SABA: Short-acting beta agonists. For non-parametric categorical and skewed continuous variables, the chi-square test, and Mann-Whitney U-test were used. Fisher’s Exact Test was used for expected counts < 5. Statistical significance was set at p < 0.05.

References

[1] Uller L, Leino M, Bedke N, et al. Double-stranded RNA induces disproportionate expression of thymic stromal lymphopoietin versus interferon-β in bronchial epithelial cells from donors with asthma. *Thorax* 2010; 65: 626–632.

[2] Du Rand IA, Blaikley J, Booton R, et al. British Thoracic Society guideline for diagnostic flexible bronchoscopy in adults. *Thorax*; 68. Epub ahead of print 2013. DOI: 10.1136/thoraxjnl-2013-203618.
